# Optimized ultrasound imaging of phase-change nanodroplets

**DOI:** 10.1016/j.ultras.2025.107933

**Published:** 2025-12-20

**Authors:** Charles R. Dyall, Dmitry Nevozhay, Andy Liu, Trevor M. Mitcham, George J. Lu, Konstantin V. Sokolov, Richard R. Bouchard

**Affiliations:** a Department of Imaging Physics, The University of Texas MD Anderson Cancer Center, 1881 East Road, Houston 77054 TX, United States; b UTHealth MD Anderson Graduate School of Biomedical Sciences, 6767 Bertner Avenue, S3.8344 Mitchell BSRB, Houston 77030 TX, United States; c Department of Bioengineering, Rice University, BioScience Research Collaborative, 6500 Main Street, Suite 1030, Houston, TX 77030, United States; d Department of Imaging Sciences, University of Rochester, 601 Elmwood Avenue Imaging Sciences - Box 648. Rochester, NY 14642-8648, United States; e Department of Biomedical Engineering, The University of Texas at Austin, 107 W Dean Keeton Street Stop C0800, Austin 78712 TX, United States

## Abstract

Phase-change nanodroplets (PCNDs) continue to generate significant research interest due to their potential to extravasate into tissue, to be targeted for molecular imaging and drug delivery, and to undergo an induced phase-change to “activated” microbubbles (MBs) for ultrasound (US) imaging. To accurately quantify molecular markers, however, one assumes a consistent proportion of PCNDs in a region of interest (ROI) are stably activated and imaged. Herein we present a framework for developing a diagnostic sequence that is optimized for PCND activation uniformity, contrast, and acquisition time. To develop this framework, activation was examined at three scales of increasing complexity: single, adjacent, and full ROI activation(s). First, transmit parameters for a single activation were optimized using PCNDs across concentrations (1.0 × 10^7^–10^9^ PCND/mL) in controlled phantom experiments, considering contrast-to-noise ratio (CNR), area, and offset (e.g., centroid axial distance from transmit focus) of the activation region along with acquisition time. Activation regions were also compared to prospective estimates based on US-beam imaging. Next, overlap and order of adjacent activation regions was optimized by maximizing spacing while preserving uniformity and minimizing signal loss from adjacent transmit interactions. Finally, an optimized raster-scanning scheme was applied to a mock tumor ROI, yielding 1.4–7.0 dB greater contrast enhancement over unoptimized schemes while reducing acquisition time. Further, PCND concentration was found to share a nonlinear relationship with US signal enhancement that differed significantly between linear and harmonic imaging modes. In conclusion, our framework’s optimization of contrast, uniformity, acquisition time, and cavitation mitigation for PCND US imaging should help facilitate its eventual clinical translation.

## 1. Introduction

Traditionally, ultrasound (US) molecular imaging has been restricted to the vasculature due to the size of microbubbles (MBs), which prevents their tissue extravasation [1] and results in their rapid elimination from blood flow [2,3]. However, as most cells of interest (e.g., cancer cells) exist outside the vascular compartment, there is need for an US contrast agent that is small enough to extravasate and has prolonged circulation in comparison to MBs [4], leading to the development of phase-change nanodroplets (PCNDs) [5,6]. This capability is particularly relevant for imaging of tumor-associated molecular markers, which can be targeted with such nanoscale contrast agents [7–9]. By enabling extravascular access, PCNDs also offer a pathway to ultrasound-based therapeutic applications, including intratumoral drug delivery [10,11], enhanced US tumor ablation [12], and local oxygenation for radiosensitization [13,14]. Although a nanodroplet’s aqueous composition does not provide a significant acoustic impedance mismatch relative to water-based tissue background, PCND formulations using perfluorocarbon (PFC) cores have been developed such that a focused acoustic transmit can “activate” the nanodroplets through the process of acoustic droplet vaporization (ADV), phase-changing them into gas-filled MBs that generate increased US contrast. As such, PCNDs can leverage the enhanced permeability and retention effect to target extravascular molecular markers, after which they can be activated to allow for conventional contrast-enhanced ultrasound (CEUS) [15–17].

Previous work has shown that CEUS signal intensity is proportional to MB concentration [18,19], allowing for quantification of hemodynamics [20] and intravascular receptor expression [21]. Thus, to achieve extravascular molecular imaging, it is critical that a consistent proportion of PCNDs be stably and uniformly activated within a region of interest (ROI). Focused US transmit pulses can be raster-scanned throughout a ROI to provide the negative pressure required for PCND activation; this can be achieved with a diagnostic imaging array to increase translational feasibility and allow for co-registered CEUS imaging of ADV-induced MBs [22].

In prior work by Dayton and his colleagues [23], the post-activation volume of PCNDs was first empirically measured in a phantom environment. For multiple activations, foci spacing was set to ensure axial/lateral overlap of these phantom-measured activation volumes to avoid gaps in ROI coverage. They also showed that transmit parameters, such as transmit pressure and cycle number, can affect critical attributes of resulting activations [24]. Building on this foundational work, we pursued optimization of “PCND imaging” (i.e., including both activation and imaging) through a stepwise approach of increasing complexity: investigating dynamics first from a single activation, then from adjacent activations, and finally from a series of activations sufficient to fully cover a tumor-like ROI. To this end, single activations resulting from PCNDs within an experimentally tractable range of concentrations were analyzed in controlled phantom geometries and optimized to maximize contrast, uniformity, and size. Additionally, US-beam imaging (i.e., beamformed backscatter from a focused transmit [25]) was examined to prospectively estimate activation regions (i.e., the region in which MBs resulting from ADV are detected), while the relationship between US signal enhancement (both linear and harmonic modes) and PCND concentration was assessed.

Once a single activation was optimized, adjacent activations (both laterally and axially) were studied to determine the optimal spacing and order of activation transmit foci. Although such overlap helps to eliminate gaps between activation regions, excessive overlap can induce destruction of previously activated MBs, leading to signal loss [26,27]. By quantifying this degradation, we were able to further fine-tune the distance between activations to reduce loss of post-activation signal. Once ideal spacing between adjacent activations was determined, performance of a group of optimally spaced activations to fully sample a mock tumor ROI was assessed. The overall goal of this work is to develop an adaptive diagnostic (i.e., under the FDA mechanical index [MI] limit) US sequence that facilitates accurate PCND identification within an imaged ROI. Such a framework for more consistent, efficient, and safe PCND imaging establishes a foundation for future preclinical and clinical validation.

## 2. Methods

### 2.1. PCND formulation

Nanodroplet synthesis was adopted from previously published studies [22,28–32]. Briefly, 14.32 mg of 1,2-distearoyl-*sn*-glycero-3-phosphocholine (DSPC, 75% by molarity) and 16.95 mg of 1,2-distearoyl-*sn*-glycero-3-phosphoethanolamine-N-methoxy-polyethyleneglycol-2000 (DSPE-PEG-2000, 25% by molarity) (both from Avanti Polar Lipids, Inc., Alabaster, AL) were mixed in chloroform (Sigma-Aldrich Corp., St. Louis, MO) and vacuum dried for 20 min in a rotary evaporator (Cole-Parmer Instrument Company, LLC, Vernon Hills, IL). The dried lipid cake was rehydrated with 2 mL of deionized (DI) water while being agitated in a rotary shaker at 300 rpm for 30 min at room temperature. Following rehydration, 200 μL of tetradecafluorohexane, 100 μL of 1% (v/v) aqueous solution of 1H,1H,2H-Perfluoro-1-hexene,3,3,4,4,5,5,6,6,6-nonafluoro-1-hexene (Zonyl PFBE) (both from Sigma-Aldrich Corp.), and 150 μL of ice-cold DI water were added directly to the vial containing the rehydrated lipids. The mixture was then vortexed for 30 sec and sonicated in ice-cold water for 1 min using an US bath (CPX-962-218R; Thermo Fisher Scientific, Waltham, MA). Finally, each suspension was sonicated with a 2-mm-tip probe-sonicator (VCX 500; Sonics & Materials, Inc., Newtown, CT) for two 1-min cycles at 25% maximum amplitude, separated by 20 sec of vortexing. The suspensions were kept on ice for 5 min before characterization.

### 2.2. PCND characterization

Dynamic light scattering (DLS) intensity distribution measurements were used to measure the size and polydispersity index of nanodroplets (Malvern Zetasizer Pro, Malvern Panalytical Ltd., Malvern, UK). The PFC content in nanodroplet preparations was measured using 19F nuclear magnetic resonance (NMR), as described in [28]. Briefly, 10 μL of sample was mixed with 75 μL of 0.5% solution of trifluoroacetic acid in deuterium oxide (both from Sigma-Aldrich Corp.) and 400 μL of deionized water. Measurements were performed in 5-mm NMR sample tubes (Wilmad-LabGlass, Vineland, NJ). The following parameters were used for NMR scans in a 500-MHz NMR spectrometer (Bruker Corporation, Billerica, MA): 241.5-ppm spectrum width; 80-ppm central frequency; 1 dummy scan; 0.58-sec acquisition time; 16 signal averages (with phase cycling); and 15-sec relaxation delay. 19F concentrations were calculated by integrating the peaks and normalizing them to a known concentration of trifluoroacetic acid used as a standard.

### 2.3. MI estimation

For MI estimation, the acoustic field simulation software Field II [33,34] was first used to model the normalized pressure resulting from an activation transmit with a 6.25-MHz center frequency (*f*_*c*_) in a plane set axially 24 mm away (30 mm lateral × 10 mm elevational; 0.1-mm and 250-MHz sampling) parallel to and centered about the array surface. A 0.2-mm NH0200 needle hydrophone (Precision Acoustics Ltd., Dorchester, UK) was then positioned in a degassed water tank 2 mm away from the face of the L11-4v transducer to measure the peak-positive pressure from a subsample of the modeled plane (19 × 5.5 mm; 1/0.5-mm axial/lateral steps; 250-MHz sampling). To generate a pressure-scaling factor, the spatial average of the hydrophone measurements was divided by a region-matched spatial average of the Field II result. The Field II result was scaled by this factor and imported as the initial condition in the Fullwave [35] nonlinear acoustic modeling software. Starting at 2 mm from the face of the transducer (i.e., the measurement plane), the pressure-scaled distribution was propagated through 14 mm of water (*α* = 0.0022 dB/cm/MHz; *β* = 3.5) and 4 mm of polyacrylamide (PAA) gel (*α* = 0.15 dB/cm/MHz; *β* = 3.5). This resulted in an estimate for peak negative pressure (*P*_*n*_) in the phantom, as done in previous work [22], which was then used to calculate $\text{MI}=\frac{P_{n}}{\sqrt{f_{c}}}$.

### 2.4. PCND imaging sequences

**Fig. 1.**
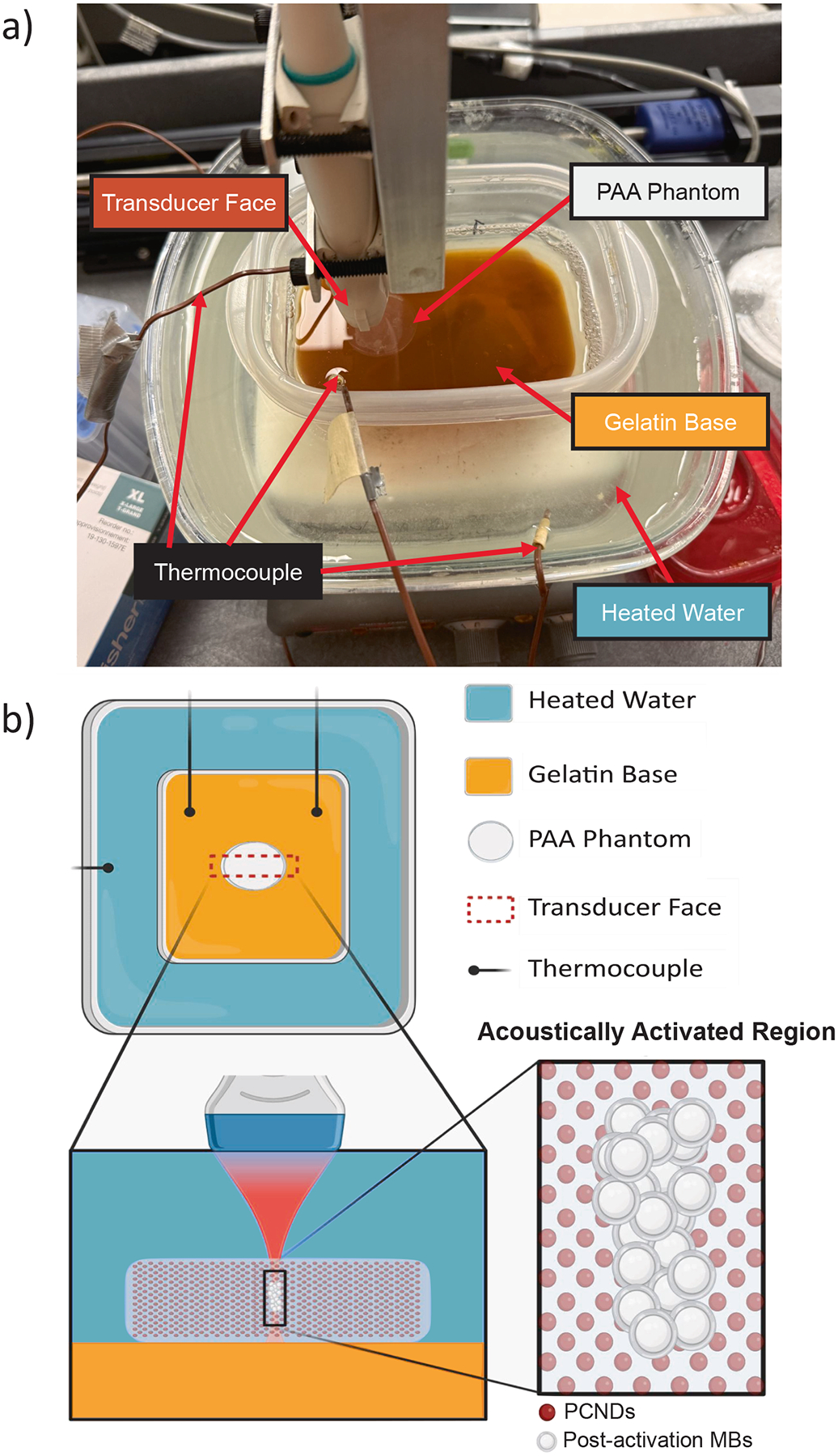
(a) Labeled photograph of experimental setup showing transducer positioned over PAA phantom in heated water tub. (b) Diagram of experimental setup (top) with zoom-in depictions of imaging-plane cross-section (bottom-left) and activation transmit in PAA phantom resulting in acoustically activated region of MBs (bottom-right) generated by phase-changed PCNDs.

All experiments were performed using an L11-4v linear array (128 elements; 6.4-MHz center frequency; 100% fractional bandwidth; 20-mm elevation focus; Verasonics Inc, Kirkland, WA) driven by a Vantage 128 US system (Verasonics Inc). The transducer was mounted to a BiSlider linear stage (Velmex Inc, Bloomfield, NY) above the phantom for translation along the elevational axis (Fig. 1a). PCND imaging sequences consisted of both focused activation (6.25 MHz; 90% relative pulse width; 1-kHz PRF; 40 V excitation) and plane-wave imaging transmits. Prior to and following delivery of activation transmits, B-mode (i.e., linear) imaging was performed using five angled plane-wave transmits (±18, ±9, 0° steering; 10.0-MHz transmit frequency; 5-kHz PRF, 67% relative pulse width; 40 V excitation, nominally 1 cycle). This was immediately followed by pulse-inversion harmonic (PIH) imaging (±18, ±9, 0° steering; 5.0-MHz transmit frequency; 5-kHz PRF, 67% relative pulse width; 40 V excitation, 1 cycle) with two plane-wave transmits delivered 180° out of phase (e.g., +/− polarity) at each angle. Prior to delay-and-sum beamforming, received RF signals for each opposite-polarity pair were summed to isolate nonlinear MB oscillation (e.g., harmonic signal). This imaging began 3 ms before commencement of activation transmits and was repeated 1 ms after activation at each focus within the field of view (FOV). For all imaging, the receive sampling rate was set to 40 MHz. For PIH imaging, a bandpass filter was applied to RF data prior to opposite-polarity pair summation using Verasonics’ InputFilter with a −3dB bandwidth of 4.47 MHz and a center frequency of 10.0 MHz.

**Fig. 2.**
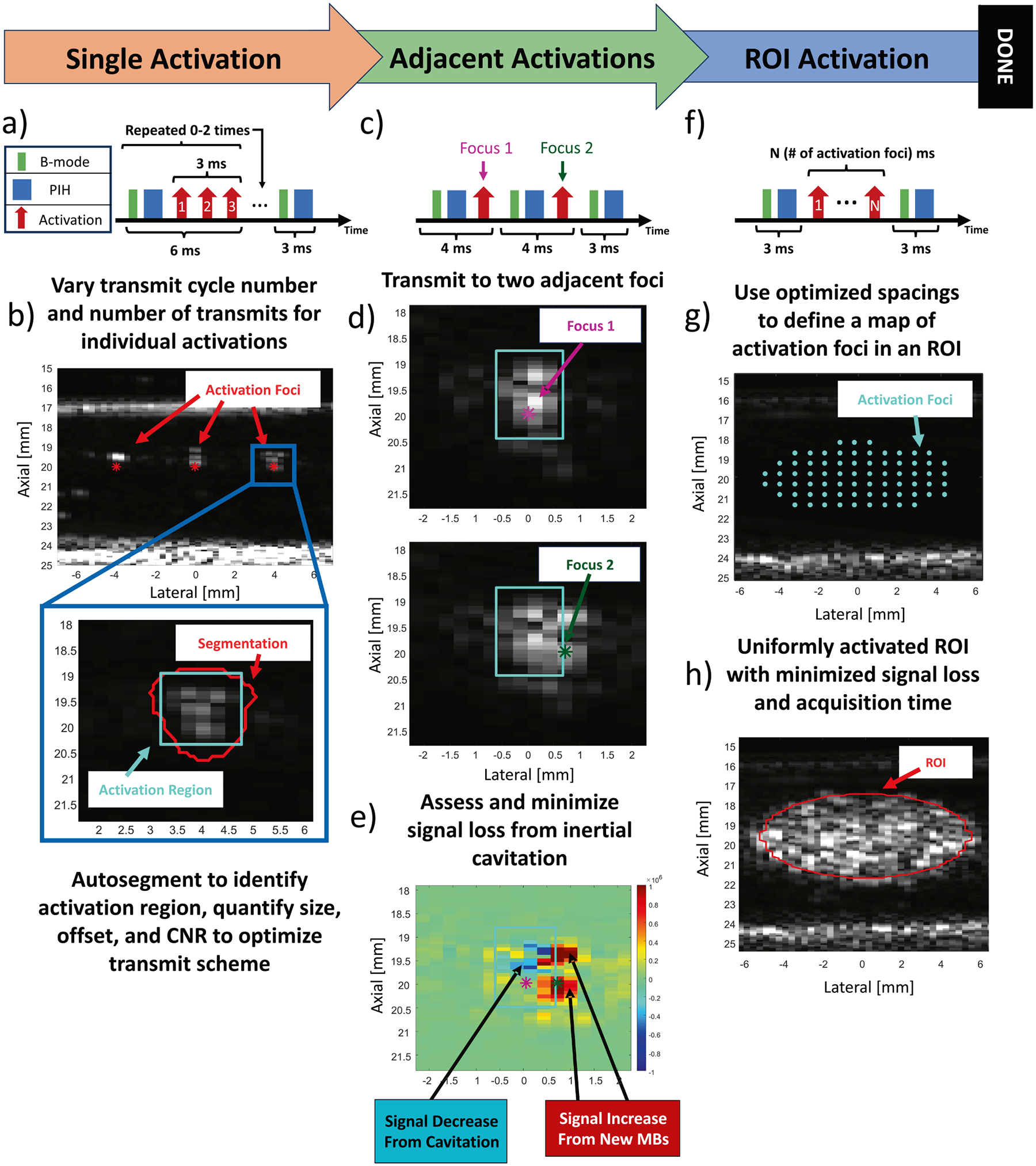
Outline of the optimization process. (a) Schematic of PCND imaging sequence and (b) result of activation transmits with segmentation (red) and activation region (cyan box) outlines. (c) The sequence for imaging adjacent activations. (d) The resulting images after each activation transmit is delivered. (e) Subtraction image showing signal loss and new signal resulting from cavitation and MB formation, respectively, from second activation. (f) Sequence for imaging ROI (red ellipse) activations with (g) activation foci (cyan dots) map generated from optimized spacings and (h) resulting post-activation ROI image. For all figures, grayscale colormap used for PIH images, while same dynamic range used for all images within the same figure unless otherwise noted.

For single-activation experiments, 3 activation foci were positioned at 20 mm axially and −4, 0, & 4 mm laterally to acquire activations in triplicate from each slice; for all experiments, activation transmits were delivered from left-to-right in the presented FOV (Fig. 2a). Transmits of 2, 4, or 6 cycles were delivered to each focus and separated by 3-ms intervals. Each transmit was repeated 3 times, with imaging acquired before and after each transmit. The effective activation transmit PRF at each focus was 167 Hz. For adjacent-activation experiments, two activations were performed with varying axial (0.25, 0.5, & 1.0 mm) and lateral (0.4, 0.725, & 1.45 mm) spacings, with the maximum spacing in each dimension based on the mean size of a single activation focus, between transmit foci in the order of left-to-right and bottom-to-top, with an extra depth-order experiment using a 0.5-mm spacing with activation performed top-to-bottom to assess the effect of near-field ADV-induced MBs on deeper activation transmits. Sequences consisted of the delivery of two adjacent activation transmits delivered at an effective PRF of 250 Hz, with B-mode and PIH imaging occurring prior to and following each activation transmit (i.e., imaging conducted in the time between adjacent transmits). For the mock-tumor (i.e., elliptical) ROI sequences, activation transmits were delivered across all foci in a raster-scan fashion from bottom-to-top. ROI activation was performed by defining a mesh grid over the image with desired activation spacing between grid points, then defining the elliptical ROI (3.5 × 9.5 mm) and selecting all the grid points within the ROI as foci for activation transmits.

### 2.5. Temperature-controlled PCND phantom

The range of tested PCND concentrations assumed that 0.1% [36] of the 1 × 10^12^ PCND/mL stock concentration (i.e., range maximum of 1 × 10^9^ PCND/mL) could be systemically delivered to a tumor. As such, 1-mL aliquots of nanodroplets were prepared via dilution in phosphate-buffered saline (1x aqueous solution; Sigma Aldrich Corp.) in a 5-mL vial to achieve final in-phantom concentrations listed with each experiment. The phantom setup is detailed in Fig. 1b. Polyacrylamide (PAA) gel phantoms were prepared by mixing 6 mL of DI water, 2 mL of 40% (w/v) acrylamide/bis 19:1 solution (GenDEPOT, Katy, TX), and 120 μL of ammonium persulfate (Sigma-Aldrich Corp.; 440-mM aqueous solution) in a 25-mL vial. This solution was then degassed for 1 min in an ultrasonic water bath, after which 60 μL of diluted PCND suspension (mixed until homogenous by repeated pipette aspiration) and 16 μL of the catalyst tetramethylethylenediamine (TEMED; Sigma Aldrich Corp.) were added and gently vortexed for 10 sec. This solution was poured into a 6-well cell culture plate (Corning Incorporated, Corning, NY) to create cylindrical phantoms (35-mm diameter × 8-mm height each). Phantoms were left in wells for 30 min to fully congeal before being removed and then adhered with cyanoacrylate glue onto the surface of a gelatin (~225 bloom, Type B Bovine gelatin; Sigma Aldrich Corp.) base in a plastic container. This phantom container was then filled with DI water and placed on a platform in a water bath heated to ~45 °C with a hot plate. Two thermocouples, positioned in opposite corners of the gelatin base, were used to monitor temperature while the hot plate was adjusted to maintain a thermocouple-averaged temperature of 37±1 °C during imaging.

### 2.6. PCND imaging protocol

**Fig. 3.**
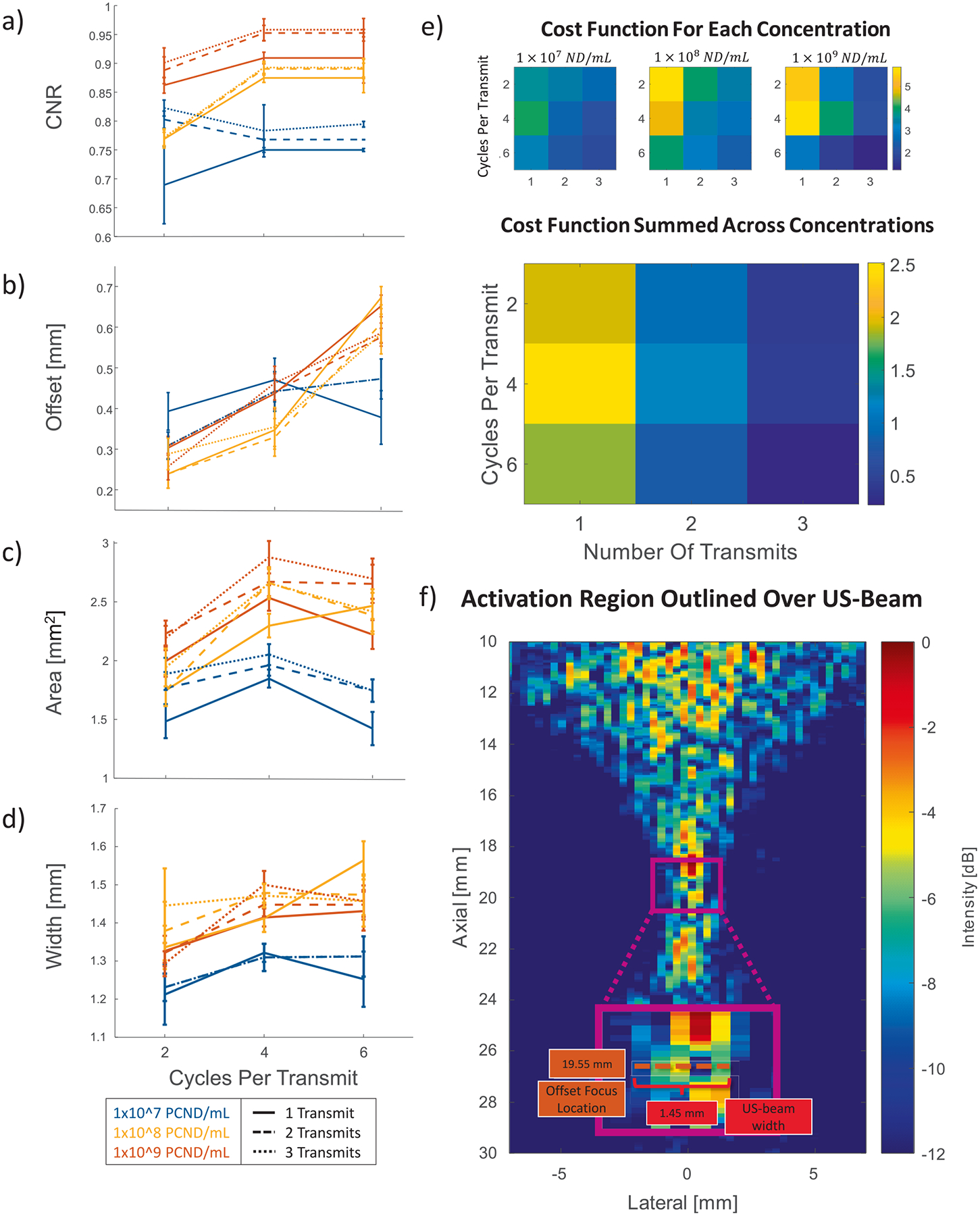
Results from single-activation experiments. Plots (mean±SE; n = 15 acquisitions) of (a) CNR, (b) offset, (c) area, and (d) width as a function of cycles per transmit. (e) Cumulative cost function values (top) averaged within each concentration (n = 15 acquisitions) as a function of cycles/transmit (y-axis) and transmit # (x-axis); cumulative summary (bottom) summed across concentration and weighted for acquisition time. (f) US-beam image of −12 dB outline, with zoom-in (magenta boxes) showing US-beam focus (orange dotted line) and US-beam width (red bracket).

An overview of the experimental workflow is provided in Fig. 2. For all experiments, the centroid of an imaged cylindrical phantom was placed at a depth of 20 mm and centered laterally relative to the US transducer face. Phantoms with concentrations of 1.0 × 10^7^, 1.0 × 10^8^, and 1.0 × 10^9^ PCND/mL were used for the single-activation experiments. The single-activation sequence (Fig. 2a) was performed for each phantom in 5 parallel imaging slices (5-mm elevational spacing) to generate 15 total activations for statistical analysis (Fig. 3a–d) and determination of an “optimized” activation transmit based on a cost function (Fig. 3e). Concentration dependence was tested using optimized activation transmits (i.e., single, 4-cycle transmit; see Results 3.2.1) and the single-activation imaging scheme (i.e., n = 15 activations) in separate PAA phantoms with concentrations of 1.0 × 10^7^, 2.0 × 10^7^, 4.0 × 10^7^, 6.0 × 10^7^, 8.0 × 10^7^, 1.0 × 10^8^, and 1.0 × 10^9^ PCND/mL, prepared by serial dilution of nanodroplets in PBS. For US-beam imaging, an optimized activation transmit centered laterally about the transducer and focused at 20 mm was imaged using reconstruction of the backscatter in a homogeneous region of a tissue-mimicking US phantom (Model 040GSE; CIRS/Sun Nuclear, Brevard County, FL).

After investigating transmit parameters for a single activation, the adjacent-activations and ROI-activation experiments were performed with optimized (i.e., based on a cost function) activation transmits in phantoms with 1.0 × 10^7^ PCND/mL, with this concentration chosen to demonstrate effective optimization in less-than-ideal PCND delivery efficiency. The adjacent-activations sequence (Fig. 2c) was performed on 15 slices for each spacing combination (Figs. 2d,e) spaced 2 mm apart in each phantom, with all initial activation transmits (e.g., “Focus 1” in Fig. 2d) being centered laterally about the transducer face and with a 20-mm focal depth. As a negative control, a “no activation” transmit (i.e., apodization zero for all elements) after the initial activation transmit was also imaged. To investigate the effect of activation depth order, an additional slice with two activations (i.e., 19.5-mm followed by 20-mm activation foci) spaced 0.5 mm axially was imaged in a top-to-bottom order. Finally, the ROI-activation sequence (Fig. 2f) was performed using three different activation tiling schemes (Figs. 2g,h) in a phantom with a concentration of 1.0 × 10^7^ PCND/mL: a wide-spaced scheme (1.0 mm axial × 1.45 mm lateral), an optimized-spacing scheme (0.5 × 0.725 mm), and a tight-spaced scheme (0.5 × 0.4 mm).

### 2.7. Image analysis

Activation regions were segmented using the Fast Marching Method seeded at the corresponding transmit focus with a foreground threshold level of 0.06 used for all data, with the threshold level determined from preliminary analysis to reliably segment activation regions from the background. Segmentation was performed on PIH post-activation images, and a rectangular activation region was created based on the median axial/lateral dimensions and centroid of the segmentation (Fig. 2b). For the single-activation experiments, segmentation was performed to outline the region of ADV-induced MBs, and the rectangular activation region was used as an analysis mask on pre- and post-activation imaging frames. Contrast-to-noise ratio (CNR), offset (i.e., axial offset of the activation region centroid from the transmit focus), area, and width (i.e., lateral dimension of activation region) were then calculated from this activation region. To consider variance in both the background and activation region, we calculated $\text{CNR}=|\frac{\bar{S_{a}}-\bar{S_{n}}|}{\sqrt{\sigma_{a}^{2}+\sigma_{b}^{2}}}$, where *S*_*a*_ is the post-activation signal in the activation region, *S*_*n*_ is the signal in a “noise” region of the post-activation frame created by translating the rectangular activation region 2 mm laterally (i.e., ensuring no overlap), and *σ*_*a*_ and *σ*_*b*_ are the standard deviation in these regions, respectively [37]. To optimize activation across all parameters for the single-activation experiment, a cost function was defined $C=\frac{\text{CNR}\times A^{2}}{\sqrt{\Delta y}}$, where *A* is area in mm^2^ and Δ*y* is offset in mm. The cost function was designed to optimize sequences in providing uniform and high-contrast coverage of a raster-scanned ROI with minimal spatial offset and transmit number, the latter reducing both acquisition time and potential for bioeffects. To this end, individual activation schemes were awarded for increased area (considered most important and thus squared) and CNR, while they were penalized for increased offset (considered least important and thus a square root applied). This was calculated for all combinations of parameters, and cumulative values were established by normalizing to the maximum value in each concentration, then summing across concentration. A weighted cost function, which penalized for acquisition time, was also calculated by dividing cumulative cost function values by $\sqrt{N}$, where *N* is number of activation transmits delivered. An “optimized” activation transmit was determined based on the maximum of the weighted cost function.

For analysis of US-beam imaging data, a binary mask was created based on a −12 dB cutoff of the reconstructed beam image; holes in this mask were then filled via a morphological closing function to account for variations from speckle. The lateral extent (i.e., “US-beam width”) and axial location (i.e., “US-beam focus”) of the narrowest region of the beam mask were determined to be the empirical beamwidth and focal point, respectively. These measurements were then compared to the mean activation region width and offset resulting from an optimized activation transmit. For concentration dependence, the post-activation image was subtracted from the pre-activation baseline to define signal enhancement resulting from ADV as $S_{E}=\bar{S_{a}-S_{b}}$, where *S*_*b*_ is the pre-activation signal within the activation region for both B-mode and PIH images. Mean interparticle distance for PCNDs was estimated by the formula $\bar{r}=\frac{1}{n^{\frac{1}{3}}}$, where *n* is the particle density (i.e., concentration). The relationship between the ratio of harmonic-to-linear signal enhancement (i.e., PIH/B-mode), interparticle distance, and concentration was also assessed.

For adjacent activations, the first activation was segmented based on its post-activation PIH image (i.e., prior to the second activation) to create a rectangular activation region in the same manner as the single-activation experiment (Fig. 2d). Subtraction images were then generated by subtracting the post-activation PIH image of the first activation from the post-activation PIH image of the second activation (Fig. 2e). Mean signal loss was calculated by summing all negative pixel values in the activation region of the subtraction image and dividing by the total signal power (i.e., total sum of pixels) of the activation region in post-activation PIH image of the first activation, then averaging (n = 15 for each spacing). Row and column pixel lines (corresponding to the electronic focus) of the final post-activation PIH imaging data (i.e., black dashed lines in Figs. 5a,b) were then extracted for lateral- and axial-spacing acquisitions, respectively, normalized to the maximum, and averaged (n = 15 for each spacing) to assess the effect of activation spacing on signal overlap/gaps. For ROI activations, the contrast enhancement was calculated as $C_{E}=\frac{S_{E}}{S_{b}}$, where pre- and post-activation masks were defined based on the ellipse used for activation foci selection but shifted 0.45 mm proximal to the transducer (Fig. 2h) to account for the US-beam focus offset (Fig. 3f).

### 2.8. Statistical analysis

A significance level of *p* < 0.05 was used for all statistical tests. Three-way ANOVA tests were used to investigate the dependence of number of transmits, cycles per transmit, or PCND concentration on CNR, area, offset, or width of resulting activation regions. Two-sample t-tests with post-hoc Holm-Bonferroni multiple comparison correction were used to determine the significance of signal loss from adjacent activations compared to the “no activation” negative control.

## 3. Results

### 3.1. PCND characterization and MI estimation

Based on DLS measurements, PCNDs had a diameter of 426±46 nm (mean±SD) and a polydispersity index (PDI) of 0.454. Based on NMR measurements, the stock PCND solution had an estimated concentration of 1.48 × 10^12^ PCND/mL. Nonlinear acoustic modeling initialized by near-field hydrophone measurements estimated *P*_*n*_ to be 3.62 MPa, which yielded an MI estimate of 1.45.

### 3.2. Single activation

#### 3.2.1. Effects of transmit Number, Cycles, and PCND concentration

Cycles per transmit significantly affected all parameters (*p* ≤ 0.0012; $n_{p}^{2}\geq 0.22$ effect size). Concentration significantly affected all parameters (*p* ≤ 0.0001; $n_{p}^{2}\geq 0.18$) except for offset, while number of transmits significantly affected area and CNR (*p* ≤ 0.0483; $n_{p}^{2}\geq 0.09$). CNR tended to increase with both increasing number of transmits and concentration while plateauing with increasing cycles per transmit (Fig. 3a). Offset tended to increase with increasing number of cycles, doubling over the evaluated range (Fig. 3b). Area tended to increase with both number of transmits and concentration, while it generally exhibited a peak at four cycles (Fig. 3c). Width, though correlated with area and following most of the same general trends, was not dependent on number of transmits (*p* = 0.6381), suggesting area growth was primarily due to increasing axial extent of activation regions (Fig. 3d). Four-cycle transmits performed best in cost function analysis (top Fig. 3e). With the acquisition time penalty of $\sqrt{N}$ applied, the weighted cost function (bottom Fig. 3e) yielded a maximum for the single, 4-cycle transmit, which was established to be the “optimized” activation transmit for this work.

#### 3.2.2. US-beam imaging

The US-beam width and focus were measured to be 1.45 mm and 19.55-mm, respectively (i.e., 0.45 mm shallow to the 20-mm electronic transmit focus; Fig. 3f). This was compared to the mean width and offset resulting from each of the single transmit schemes of two, four, and six-cycles across all concentrations. These schemes resulted in activation widths (mean±SD) of 1.15±0.31, 1.39±0.09, and 1.29±0.29 mm, respectively, and offsets of 0.32±0.18, 0.41±0.21, and 0.55±0.25 mm, respectively. A single, 4-cycle transmit was found to be the optimized transmit scheme for PCND activation as it was the best performing based on cost function analysis (Fig. 3e) and had width/offset metrics most similar to those obtained from US-beam imaging, with differences between the two less than 1 SD of corresponding activation measurements.

#### 3.2.3. Concentration dependence

**Fig. 4.**
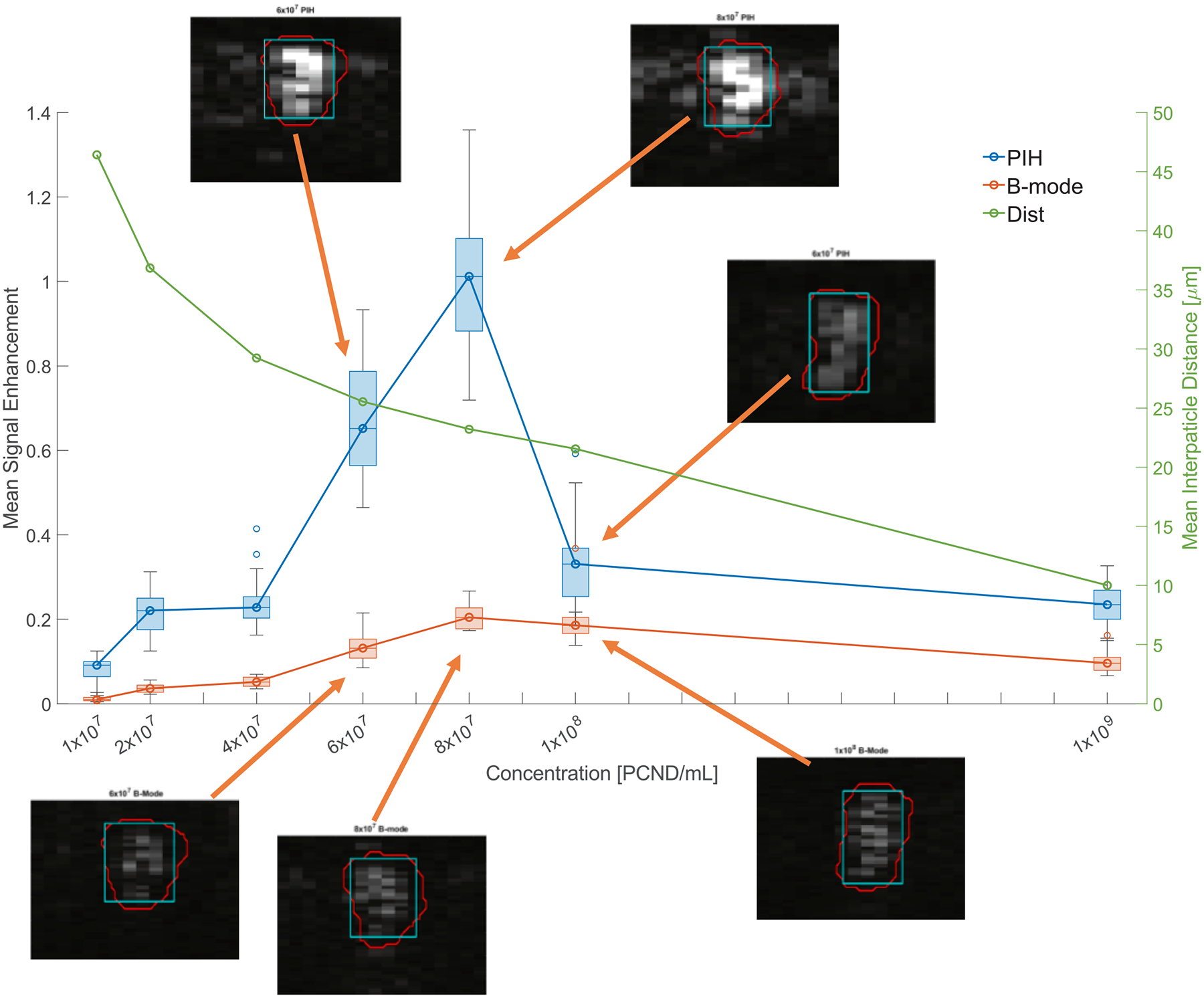
Mean signal enhancement over concentrations tested using serial dilution of PCNDs. Shown is the normalized mean signal enhancement within both PIH (blue) and B-mode (orange) activation regions for concentrations spanning 1×10^7^ to 1×10^9^ PCND/mL with representative post-activation images for PIH (top, same dynamic range) and B-mode (bottom, same dynamic range) at points of interest, along with the mean interparticle distance (green) for PCNDs in each concentration.

Based on the mean signal enhancement (n = 15) versus PCND concentration data (Fig. 4), both B-mode and PIH imaging modes presented with two distinct regions: a lower-concentration one (1.0 × 10^7^ −8.0 × 10^7^ PCND/mL) where signal increased linearly (>10x over this range) with concentration and a higher-concentration one (8.0 × 10^7^ −1.0 × 10^9^ PCND/mL) where it monotonically decreased. While B-mode signal tended to decrease linearly across this higher-concentration range, PIH signal decreased nonlinearly, presenting with a drastic >3x decrease from 8.0 × 10^7^ to 1.0 × 10^8^ PCND/mL, which corresponds to a mean interparticle distance of ~20 μm.

### 3.3. Adjacent and full-ROI activations

Mean signal loss and two-sample *t*-test (i.e., against the negative control, which presented with a mean loss of 7.1%) p-values were found to decrease with increasing distance between activation transmits. An initial threshold of *α*_0_ = 0.05 was used for significance and altered via Holm-Bonferroni multiple comparison correction for each test, with p-values higher than the adjusted significance threshold indicating non-significant findings. For axial spacings, 0.25-mm spacing resulted in 10.1% mean signal loss (*p* = 0.02), 0.5-mm spacing in 9.4% loss (*p* = 0.13), and 1.0-mm spacing in 9.2% loss (*p* = 0.17). For lateral spacings, 0.4-mm spacing resulted in 11.2% signal loss (*p* = 0.0014), 0.725-mm spacing in 7.8% loss (*p* = 0.52), and 1.45-mm spacing in 7.1% loss (*p* = 0.95). After Holm-Bonferroni correction, all spacings except for the 0.4-mm spacing were found to lack significant signal loss.

**Fig. 5.**
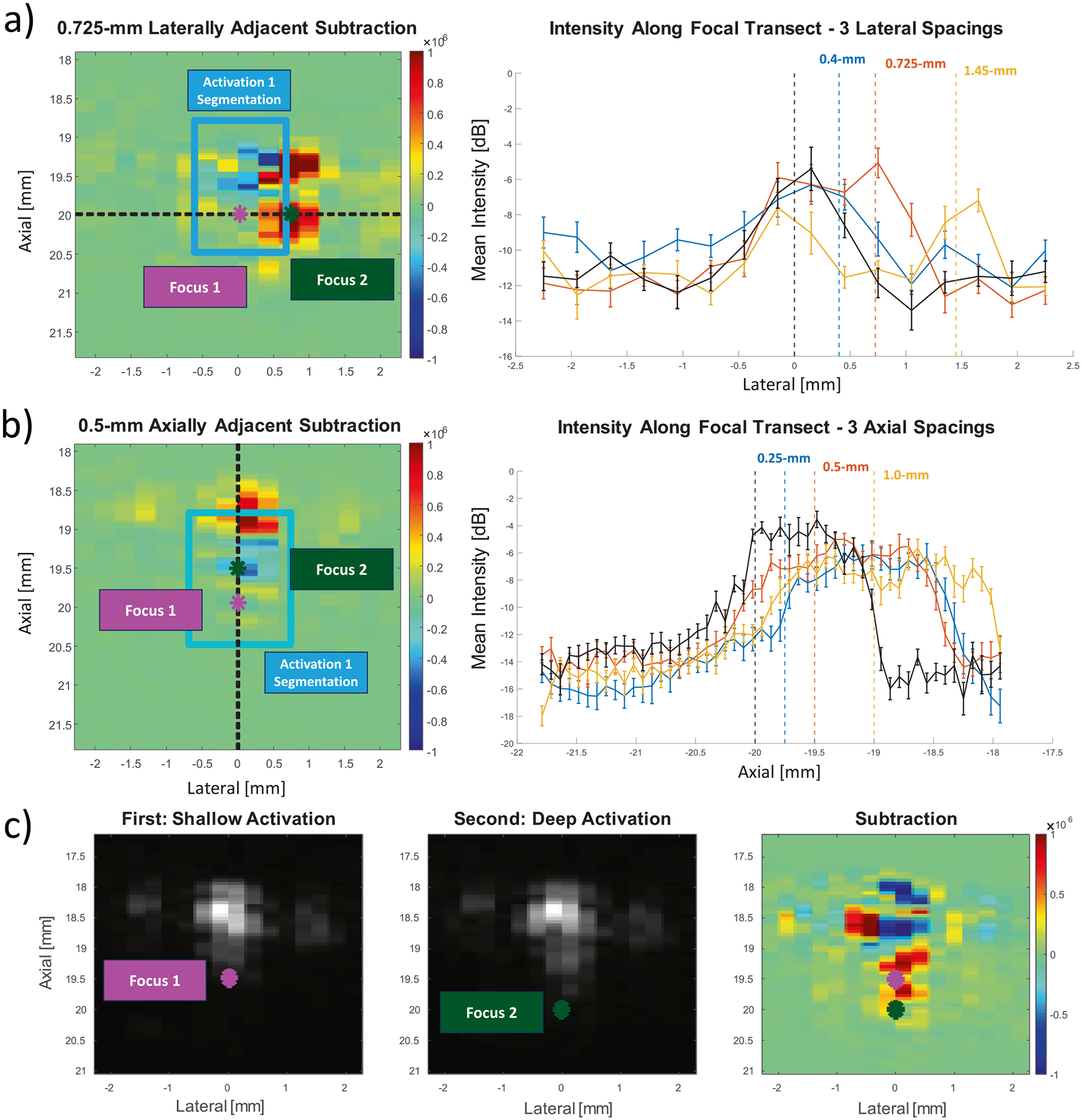
Results from adjacent-activation experiments. (a) Subtraction image (left) of 0.725-mm laterally adjacent transmits (magenta asterisk = first activation; green = second). Plots (right; mean±SE; n = 15 acquisitions/spacing) of final post-activation intensity along horizontal focal transect (black dashed line) for second-activation lateral spacings of 0.4 (blue), 0.725 (orange), and 1.45 mm (yellow); transect intensity following first activation (black) provided as baseline. (b) Subtraction image (left) of 0.5-mm axially adjacent transmits and plot (right) post-activation intensity along vertical focal transect for second-activation axial spacings of 0.25 (blue), 0.5 (orange), and 1 mm (yellow); first-activation baseline (black) also provided. (c) Demonstration of depth-order effect. Post-activation PIH images of shallower activation (left-most; 19.5 mm) followed by deeper activation (center image; 20 mm). Subtraction image (right-most) demonstrates significant near-field cavitation (dark blue signal from ~18–19 mm) that results by activating shallow-to-deep (i.e., instead of optimized deep-to-shallow ordering).

**Fig. 6.**
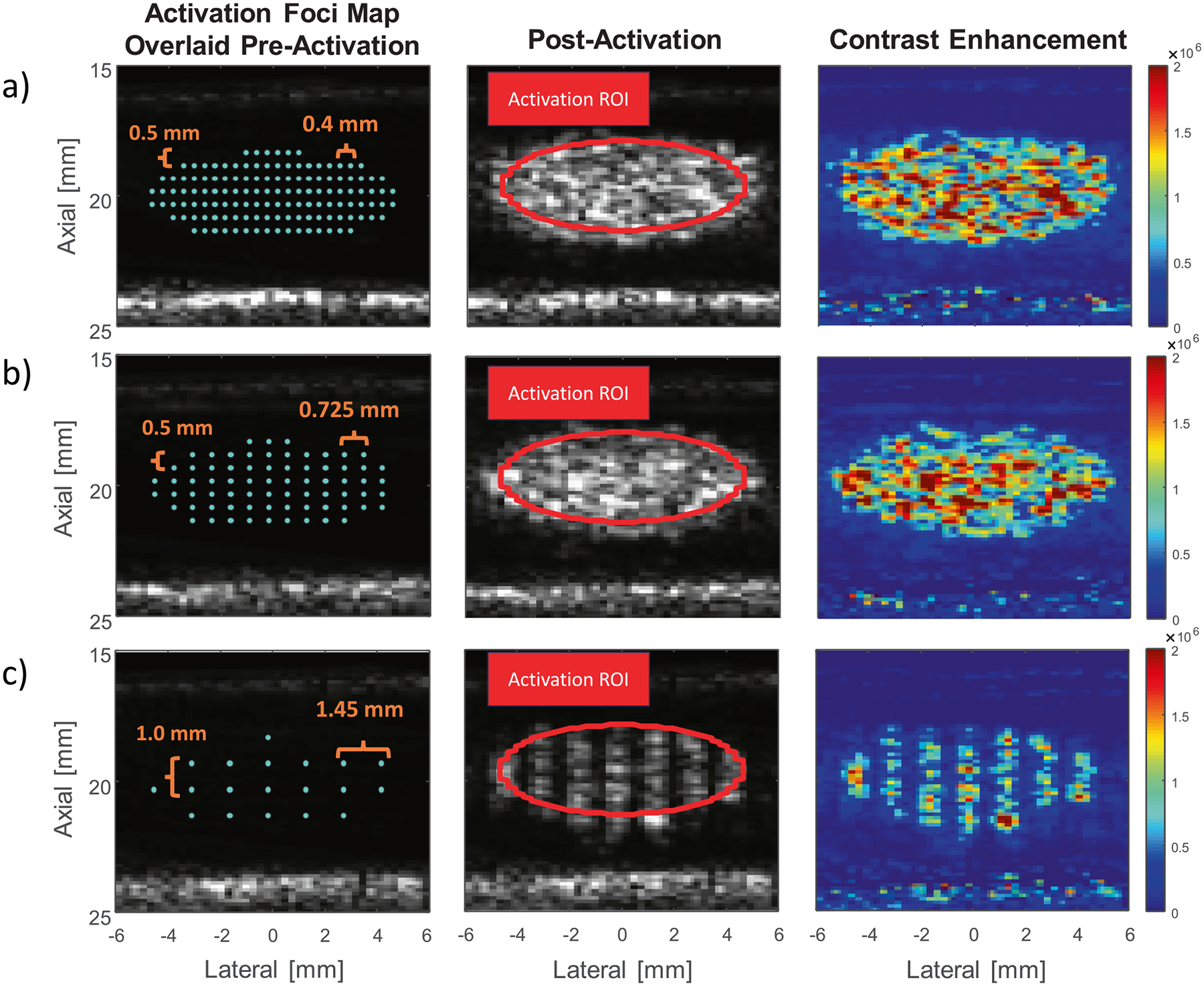
Results from ROI-activation experiments. (a) Activation foci map overlaid pre-activation image (left), post-activation image (center), and contrast enhancement image (right) of tight-spaced scheme. (b) The resulting activation of an optimized scheme, demonstrating the highest contrast enhancement. (c) The resulting activation of a wide-spaced scheme, showing gaps between the activated regions and low contrast enhancement. Images of the same type shown with matching dynamic ranges.

Activation with wide spacing resulted in gaps between activation regions consistently for both axial and lateral spacings. In the other extreme, tight spacing, aside from being inefficient (i.e., requiring more activation transmits), also demonstrated increased signal loss from the initial activation region. This was also demonstrated in the depth-order experiment, which produced a mean (n = 6) signal loss of 12.2% (Fig. 5c). The optimal lateral spacing was found to be 0.725 mm (half the US-beam width) and the optimal axial spacing was found to be 0.5 mm (~2/3 the lateral spacing). For the ROI-activation experiments, contrast enhancement was measured for each tiling scheme (see Methods 2.6). The tight-spaced scheme resulted in contrast enhancement of 81.2±43.7 (+5.5 dB; Fig. 6a), the optimized scheme resulted in contrast enhancement of 115.7±70.9 (+7.0 dB; Fig. 6b), and the wide-spaced scheme resulted in contrast enhancement of 22.9±21.1 (Fig. 6c).

## 4. Discussion

An optimized PCND imaging sequence is a careful balancing act between maximizing activation coverage and minimizing the risk of cavitation-induced signal loss. This is dependent on the size of the activation region and the spacing between adjacent foci in the activation scheme. Our framework allows for the development of optimized schemes on any programmable US device. Optimizing the sequence in this manner facilitates uniform coverage with a minimal number of transmits, reducing both acquisition time and potential for bioeffects.

It is also important to consider the effect of droplet size heterogeneity on activation dynamics. The PDI (0.454) of the PCNDs in our study indicates a broader size distribution, which can influence ADV thresholds and bubble stability. Smaller droplets generally require higher pressures for activation, whereas larger droplets undergo ADV more readily [38,39]. Such variability may lead to nonuniform activation within the ROI, particularly at lower transmit pressures, and could account for discrepancies observed between matched acquisitions and/or have affected concentration-dependent results (e.g., inclusion of larger PCNDs, making coalescence more likely at high concentrations). While our optimization framework mitigates these effects by tuning transmit parameters for consistent activation, we acknowledge that polydispersity introduces variability that warrants further investigation.

Although the weighted cost function yielded an optimized activation with one transmit, both CNR and area were shown to increase – albeit modestly in many cases (Figs. 3a,c) – with increasing transmit number, suggesting that a scheme consisting of repeated transmits to each focus may be ideal for optimizing image quality alone (Figs. 5a,c). The growth of the activation region in the axial direction with increasing transmit number is likely due to an increased probability of ADV associated with repeated activation attempts. The resulting increase in ADV-induced MBs in the near-field further attenuates the activation transmit, limiting such growth in the far-field. Since an activation transmit exhibits a comparatively sharp pressure gradient in the later extent near its focus, the activation region does not tend to grow substantially in this dimension. However, the increased acquisition time and potential for cavitation-induced signal loss can be significant drawbacks in a clinical setting, and thus the potential impact (and benefit) from repeated transmits warrants further investigation. Additionally, offset was found to increase with transmit cycle number (Fig. 3b), suggesting that increased activation volume in the near-field from higher cycle numbers has a “shielding” effect, attenuating the transmit such that deeper regions fail to activate [39]. It is also possible that alternative weightings for cost-function components may yield different optimal parameters.

In the concentration dependence experiment, signal enhancement exhibited relatively linear increase up to a concentration of 8.0 × 10^7^, demonstrating a consistent proportion of PCNDs were converted to MBs across concentrations. However, enhancement decreased in higher concentrations, potentially due to shielding from near-field MBs [27]. The sharp, nonlinear decrease in enhancement for PIH images may correspond to bubble coalescence, as the interparticle distance (green plot in Fig. 4) starts to approach the size range of post-activation MBs (estimated 1–10 μm) [40]. Coalescence may cause MBs to be further from the optimal size for nonlinear resonance. Because of this change in relative signal strength between the two imaging modes, these findings suggest the linear-to-harmonic ratio may be useful for PCND quantification, warranting future investigation.

In adjacent-activation experiments (Figs. 5a,b), wider spacing resulted in large gaps between activation regions, while the tightest spacing generated a uniform activation region but incurred significant signal loss within the initial activation region. This is consistent with the ROI-activation experiment, where the wide-spaced scheme produced the lowest contrast enhancement and visible gaps throughout ROI. Although the optimized and tight-spaced schemes produced qualitatively similar results (Fig. 6), the optimized scheme afforded the highest contrast enhancement (i.e., 1.4 dB over the tight-spaced scheme). The decrease in contrast (and durable activation signal enhancement) with the tight-spaced scheme may be an indicator of cavitation-induced signal loss resulting from activation transmits on adjacent (previously activated) MBs. This was supported by the greatest signal loss being experienced in the depth-order experiment (Fig. 5c), which implemented a top-to-bottom order, resulting in MBs in the near-field that were subject to cavitation upon activation transmits to deeper, subsequent foci. Additionally, optimal activation spacing may be prospectively estimated with US-beam imaging due to the similarity in their characterization parameters (i.e., width and focus/offset; Figs. 3b,d,f). This can be achieved by using the optimized transmit to acquire a pilot US-beam image in a homogenous tissue region. The lateral spacing may then be estimated as half the US-beam width, with axial spacing defined as less than or equal to the lateral spacing to ensure adequate overlap, mitigating gaps. The US-beam focus also effectively estimates the activation region offset. As such, US-beam imaging *in situ* may provide a starting point for adaptation, though *in vivo* heterogeneity will require additional calibration.

Our framework’s clinical translation potential can be improved by delivering uniform pressure across all activation foci. As our ROI was a relatively small portion of the FOV that was centered about the transducer’s elevational focus, we did not consider this to be a significant drawback in the presented work. However, activation pressure matching [24] could be incorporated to achieve a more consistent distribution of post-activation MBs in larger ROIs. Another limitation of this work is the examination of only one nanodroplet formulation. As dynamics of PCND activation are highly dependent on formulation, future work will be needed to examine *in vitro* behavior of other PCND formulations and recalibrate activation parameters and spacing accordingly [6,26,41]. While the estimated mechanical index of 1.45 for activation pulses is below the FDA diagnostic limit of 1.9, it could be considered too high for certain clinical applications (e.g., ophthalmic imaging), and thus further investigation is needed to assess the relationship between lower-MI sequences and image quality. Additionally, although PCND concentrations spanning two orders of magnitude were assessed, *in vivo* delivery yields can vary substantially and with significant spatial heterogeneity [36]. Therefore, expanded concentration ranges (particularly at even lower concentrations) along with nonuniform phantoms should be explored in future work. Lastly, although developed with the intention of improving US molecular imaging, this sequence also exhibits strong potential in improving PCND-mediated drug delivery [5,6]. As there is a similar need to activate PCNDs uniformly within a given ROI to ensure consistent drug payload release, this framework can improve safety and efficiency. This framework can then be used to develop optimized sequences that utilize targeted, drug-loaded nanodroplets for theranostic studies.

## 5. Conclusion

Currently, US molecular imaging is restricted to the vasculature due to MBs being unable to access extravascular tumor regions. PCNDs have demonstrated strong potential as an extravascular US contrast agent, but there are many factors to consider in activating and quantifying the presence of generated MBs. The resulting activation size and spacing between adjacent activations can be estimated prospectively via US-beam imaging to ensure uniformity and reduce the risk of cavitation-induced signal loss. Raster-scanning using optimized activation transmits and spacing results in a sequence that uniformly activates a desired ROI while minimizing acquisition time and bioeffect risks. This preliminary *in vitro* work establishes a foundation for future implementation in heterogeneous tissue and *in vivo*. Such benefits make this framework a useful platform for the study of PCNDs toward their translation into clinical US molecular imaging.

## Data Availability

Data will be made available on request.

## Glossary

PCNDs: Phase-change nanodroplets
ADV: Acoustic droplet vaporization
MB: Microbubble
DI: Deionized
CNR: Contrast-to-noise ratio
C6: Perfluorohexane
PIH: Pulse-inversion harmonic (imaging)
ROI: Region of interest
ANOVA: Analysis of variance
TEMED: Tetramethylethylenediamine
PFC: Perfluorocarbon
DLS: Dynamic light scattering
NMR: Nuclear magnetic resonance (imaging)
FDA: Food and Drug Administration
CEUS: Contrast-enhanced ultrasound
